# Prevalence and impact of sickle cell trait on the clinical and laboratory parameters of HIV infected children in Lagos, Nigeria

**DOI:** 10.11604/pamj.2018.31.113.15097

**Published:** 2018-10-15

**Authors:** Agatha Nkiruka David, Munirah Yewande Jinadu, Agatha Eileen Wapmuk, Titilola Abike Gbajabiamila, Jane Ogoamaka Okwuzu, Ebiere Clara Herbertson, Oliver Chukwujekwu Ezechi

**Affiliations:** 1 Department of Clinical Sciences Nigerian Institute of Medical Research 6 Edmund Crescent, Yaba, Lagos, Nigeria; 2 Department of Community Health, Federal Medical Centre, Ebute Meta, Lagos Nigeria

**Keywords:** HIV, children, sickle cell disease, sickle cell trait, Nigeria

## Abstract

**Introduction:**

sickle cell disease and HIV infection are prevalent in sub-Saharan Africa. While Haemoglobin S (HbS) contributes to significant morbidity and mortality in the homozygous or double heterozygous states, in the carrier state it confers a survival advantage in disease conditions such as malaria. However the interaction between sickle haemoglobin and HIV infection, especially in children remains largely unknown. This study aimed to assess the prevalence and impact of sickle cell trait on the clinical and laboratory parameters of HIV infected children in Lagos, Nigeria.

**Methods:**

a cross-sectional study among HIV infected children in an HIV treatment centre in Lagos, Nigeria. Socio-demographic and clinical characteristics were obtained and blood sample collected for haemoglobin electrophoresis, HIV RNA viral load and haematologic profile. Data was analysed with SPSS version 20.

**Results:**

the prevalence of sickle cell trait was 18.8% among the 208 study participants, with none having sickle cell disease (SCD). Participants with SCT were significantly younger (OR = 4.0 95% CI (1.74-9.24)), more likely to be from the Yoruba ethnic group (OR = 3.3 95% CI [1.45-7.52)), had more opportunistic infections (OR = 2.4 95% CI (1.18-5.03), and lower mean HIV RNA viral load (p = 0.05) at baseline. However response to HIV care and treatment was similar in both groups of participants.

**Conclusion:**

the finding of absence of SCD, low prevalence of SCT, and lower HIV viraemia in HIV infected children with SCT may have implications for childhood survival which requires further clarification in future studies.

## Introduction

Sickle cell disease (SCD), an autosomal recessive disorder caused by the inheritance of abnormal sickle haemoglobin (Hb) S or C from both parents, is among the commonest severe genetic disorders globally [1]. It results from a mutation in the β haemoglobin gene that causes haemoglobin polymerisation in the red blood cells (RBC) in situations of low oxygen tension. In the homozygous state (Hb SS or HB SC), this leads to rigidity of the rbc, a change from the normal discoid to a sickle shape, and clumping together of the cells especially in small capillaries. This lead to vaso-occlusion which is the hall mark of the disease, resulting in haemolytic anaemia, bone pain crises, increased susceptibility to infections especially with encapsulated organisms like streptococcus pneumoniae, and end organ dysfunction [2]. It is estimated that approximately 300,000 children are born every year with SCD in the world, out of which over 75% are found in Sub-Saharan Africa [3, 4]. In Nigeria, the prevalence of SCD is 20-30/1000 live births [5]. The burden of the disease has reached a level where it contributes 9-16% to under-five mortality in many West African countries [6]. Hemoglobinopathies alone represent a health burden comparable to that of communicable and other major diseases [7]. The inherited sickle haemoglobin gene may also present in a carrier state when paired with normal HbA for a genotype HbAS or HbAC, described as “sickle cell trait” (SCT). Though individuals with the sickle cell trait usually present with no, or very mild symptoms, life threatening complications such as gross haematuria, splenic infarction and exertional heat illness can occur in cases of hypoxia due to the polymerization of the deoxy haemoglobin S [8]. The prevalence of the sickle cell trait in many tropical African countries including Nigeria ranges between 20 and 30% of the population [9-12]. SCT is common in regions with high malaria endemicity as it confers a survival advantage against malaria [3, 4]. Simultaneously, the human immunodeficiency virus (HIV) is also endemic in sub-Saharan Africa (SSA) where 75% of people affected with SCD are to be found. The World Health Organization (WHO) estimates that 23.5 million of the world's 34 million people living with HIV/AIDS and 90% of the world's 3.4 million HIV-infected children live in SSA [13, 14]. The frequent occurrence of haemolytic anaemia and risk of cerebrovascular disorders in SCD make repeated blood transfusions a recognized management strategy for this condition [1].

On the other hand, although blood transfusion is diminishing in importance as a means of HIV transmission in the face of improved screening methods and ever diminishing window period, it remains an important mode of transmission of HIV, especially in remote, resource limited areas. Thus a high prevalence of comorbidity with HIV and SCD is expected in populations in tropical Africa. However, some reports from within and outside Africa suggest a lower prevalence of HIV in persons with SCD, in contrast to Hepatitis B and C which share similar routes of transmission with HIV but have higher prevalence in persons with SCD [14-18]. Malaria, another endemic disease in tropical Africa, shows a markedly reduced incidence in persons with SCT while the disease is devastating in those with SCD. It has in fact been postulated that the sickle cell gene evolved as a protective gene against malaria parasites, being most common in areas of high malaria endemicity, with the carrier state (SCT) conferring a survival advantage [3, 19-21]. Other genetic conditions have also been found to confer resistance to some infectious diseases; notable are cystic fibrosis and cholera, Tay-Sachs and tuberculosis and Myasthenia gravis and rabies [22]. There may also be a complex interaction between SCD and SCT and HIV which requires further study for elucidation. It has been postulated that the chronic hypoxia and generation of inflammatory cytokines that are a hallmark of SCD may be detrimental to the replication of the human immunodeficiency virus thus making co-morbidity with the two disease conditions a rarity [15]. The study by Kunari and colleagues also showed lower HIV infection rate among people with sickle cell trait compared to the general African-American population. Their findings revealed that some iron- and hypoxia-regulated host factors responsible for HIV-1 replication were altered in SCT [23]. A review of the role of cellular iron and oxygen in the regulation of HIV-1 infection by Nekhai and colleagues [24] showed that in situations of hypoxia, there are reduced CDK9 activity and reduced HIV transcription and replication. There were also significantly lower expressions of HIV-1 *env* and *gag* mRNA in HIV-1 infected persons with SCT [23]. All these may explain the deregulation of HIV-1 infection in persons with SCT. While there have been a number of studies assessing the prevalence of HIV among persons with SCD and SCT, there is paucity of data assessing SCD and SCT in persons infected with HIV, especially in Nigeria, a country that is endemic for both conditions. The present study was conducted to determine the prevalence of sickle haemoglobin and its impact on clinical, haematological and virologic parameters in HIV infected children 1 to 14 years in Lagos, Nigeria.

## Methods

**Study area:** this study was carried out at the HIV care and treatment Centre of the Nigerian Institute of Medical Research (NIMR), Yaba, Lagos, Nigeria. The treatment centre was established in 2002 to provide research backup for the Federal Government of Nigerian's Antiretroviral Drug Access Initiative. The centre currently provides comprehensive HIV treatment, care and support to more than 24,000 patients, including adults, children and pregnant women, with diverse backgrounds in terms of ethnicity, religion, socio-economic status and culture. Majority of the patients come from Lagos and other states in the southwest geopolitical zone of the country, with a few coming from the rest of Nigeria and neighbouring West African countries.

**Study design:** this was a cross sectional study conducted between March and July, 2017 among confirmed HIV infected children aged 1 to 14 years receiving comprehensive HIV care and support at the Child and Adolescent Clinics of the Treatment Centre. Patients who were very ill, aged more than 14 years, or declined consent/assent were excluded from the study. Consenting randomly selected respondent caregivers were interviewed with a semi-structured questionnaire to obtain socio-demographic and HIV related information. After interview, 5 ml of venous blood was collected from participants and used to determine the Hb genotype, current haemoglobin (Hb) at the Clinical Sciences Research Laboratory and CD4 count and HIV RNA viral load at the Human Virology Laboratory. Hb was determined using the Sysmex haematology autoanalyser (Sysmex.co.za), CD4 with the Cyflow Counter (Sysmex.co.za), and HIV RNA viral load with the Roche Amplicor (Roche Molecular Systems). Hb electrophoresis to determine genotype was done using the cellulose acetate buffer method. The sickling status of each sample was determined by adding one to two drops of blood sample onto a tile. The sample was diluted with distilled water to lyse cells. The lysed cells were transferred to numbered sample holders and with the aid of sample applicator was transferred onto the cellulose acetate paper. Samples were applied to cellulose acetate agar gel and haemoglobins were separated by electrophoresis using an alkaline buffer (Tris-EDTA with Boric Acid) at pH 8.4. Each haemoglobin variant carries a different net charge so they migrate at varying speeds. The machine was set at 250V and the separation was read after 20 minutes. Following electrophoretic migration, visualization of hemoglobin bands was accomplished and compared with known standards. This method yields rapid and reproducible separation of HbA, HbF, HbS and HbC as well as other variant hemoglobins with minimal preparation time.

**Data analysis:** data were entered into excel spreadsheets, cleaned and analysed using SPSS version 20 (SPSS Inc, Chicago). Frequency tables were computed for all variables. The chi square test and student t-test were used to test for association between categorical and continuous variables respectively. Binary logistic regression was used to derive odds ratios and statistical significance was set at p value < 0.05.

**Ethical considerations:** ethical approval for the study was obtained from the Institutional Review Board (IRB) of the Nigerian Institute of Medical Research, Lagos, Nigeria (IRB/17/011). Written informed consents and assents were obtained from caregivers and children respectively for the study. Children for whom consent and/or assent could not be obtained were excluded from the study, but continued to receive appropriate clinical care.

## Results

**Figure 1:**
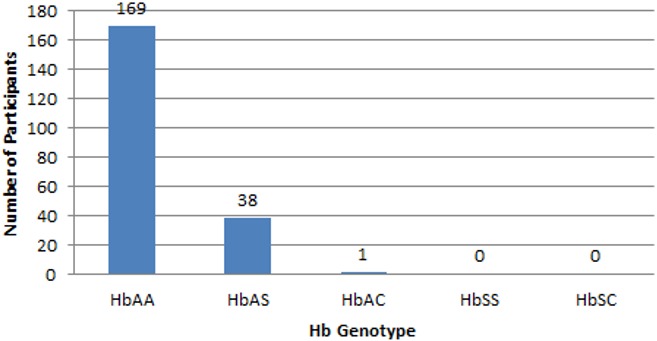
distribution of HB genotypes among participants

Table 1 shows the sociodemographic characteristics of the 208 study participants. The mean age of participants was 8.6 ± 3.2 years. Those aged less than 10 years were in the majority (54.8%). There were 108 males and 100 females (M: F = 1.1:1). Majority of the participants were in primary school (127 (61.1%)), and belonged to the two major ethnic groups in Southern Nigeria (Igbo (42.8%), Yoruba (30.3%)). Caregivers completed secondary school in majority of cases (66.8% and 65.8% for fathers and mothers respectively). The baseline clinical and laboratory characteristics of study participants are shown in Table 2. The mean age at HIV diagnosis, weight for age (for participants younger than 5 years)/body mass index (BMI) (for participants aged 5 years or more) Z score, and WHO clinical stage were 3.2 (± 2.7) years, -1.02 ± 1.27, and 2.2 ± 0.8 respectively. The mean HIV RNA viral load, CD4 count and Hb were 727,611.6 (± 1,572,783.5) copies/mL, 886.9 (± 691.6) cells/μL, and 9.4 (± 1.4) g/dL respectively. Most participants (97.1%) were on antiretroviral drugs (ARVs) (80.8% on first line and 16.3% on second line ARVs)), with a mean duration on ARV of 6.3 (± 2.9) years. Figure 1 shows the distribution of Hb genotypes among study participants. There were 169 (81.3%), 38 (18.3%), and 1 (0.5%) participants with HbAA, HbAS, and HbAC, respectively. None of the participants had HbSS or HbSC. The prevalence of sickle cell trait (SCT) (i.e HbAS and HbAC) and SCD (HbSS and HbSC) from this study was therefore 18.8% and 0.0% respectively. Table 3 shows the comparative analysis of socio-demographic and clinical characteristics between participants with SCT and those with normal HbAA. There was no significant difference in the sex ratio among those with sickle cell trait (SCT) and those with HbAA (0.9:1 versus 1:1.1 respectively; p = 0.424). At the time of HIV diagnosis and enrollment into care and treatment, mean age (SCT: 3.0 (± 2.8) years; HbAA: 3.3 (± 2.8 years), mean weight for age/BMI Z score (SCT: -1.2 (± 1.1); HbAA: -0.98 (± 1.3)), and mean WHO clinical stage (SCT: 2.4 (± 0.8); HbAA: 2.2 (± 0.9)) were similar in the 2 groups. SCT was however significantly associated with being from the Yoruba ethnic group (51.3% versus 35.5% (OR = 3.3 (95% CI 1.45-7.52), p = 0.003]), and current age younger than 10 years (79.5% versus 49.1% (OR = 4.0 (95% CI 1.74 -9.24), p < 0.001)). Duration on ART and type of ARVs were similar between the two groups. There was no significant difference in the history of blood transfusion (7.7% versus 13.0%), jaundice (7.7% versus 4.1%), and bone or joint pains (2.6% versus 3.6%) among participants with SCT and those with HbAA respectively. However, history of TB (as a hallmark of opportunistic infections in HIV) was significantly more common in participants with SCT (56.4% versus 24.1% (OR = 4.08 (95% CI 1.97-8.43), p < 0.001)). This is depicted in Table 4. Table 5 shows that there was statistically significant lower baseline mean HIV RNA viral load among participants with SCT than those with HbAA (415,384 (± 545,026.7) versus 812,764 (± 1744181.6) copies/ml; p = 0.048). Though the mean CD4 count and Hb levels were lower in the participants with SCT, this was not statistically significant. The mean current HIV RNA viral load was also lower among participants with SCD (11,656.5 (± 34,152.9) versus 42,834.6 (± 333679.0) but this was also not statistically significant. The current mean CD4 count and Hb level were similar in the two groups of participants.

**Table 1:** socio-demographic characteristics of respondents

Characteristic	Number of participants (%) n = 208
**Age (years)**	
1-9	114 (54.8)
10-14	94 (45.2)
Mean ±SD	8.6 ± 3.2
**Sex:**	
Male	108 (51.9)
Female	100 (48.1)
**Ethnic Group:**	
Yoruba	63 (30.3)
Igbo	89 (42.8)
Hausa	9 (4.3)
Others	47 (22.6)
**Class:**	
Pre-School	40 (19.2)
Primary School	127 (61.1)
Junior Secondary	36 (17.3)
Senior Secondary	5 (2.4)
**Parental Education:**	
**Father (n = 181):**	
< Secondary	42 (23.2)
≥ Secondary	139 (76.8)
**Mother (n = 179):**	
< Secondary	42 (23.5)
**≥** Secondary	137 (76.5)

**Table 2:** baseline clinical and laboratory characteristics of participants

Characteristic	Mean (± SD)
Age at HIV Diagnosis (years):	3.2 (±2.7)
BMI/Weight for Age Z Score	-1.02 (±1.27)
Who Clinical Stage at Diagnosis	2.2 (±0.8)
Baseline HIV Viral Load (copies/ml)	656,183.0 (±1,324,159.2)
Baseline CD4 Count (cells/μL):	886.9 (± 691.6)
Baseline Hb (g/dL):	9.4 (±1.4)

**Table 3:** relationship between socio-demographic and HIV Characteristics among HbAA and SCT

Characteristic	SCT (n = 39)	HbAA (n =169)	OR (95% CI)	P value
**Sex:**				
Male	18 (46.2)	90 (53.3)	0.75(0.37-1.51)	0.424
Female	21 (53.8)	79 (46.7)	1.0	
**Current Age Group (years):**				
1 - 9	31 (79.5)	83(49.1)	4.0 (1.74 – 9.24)	**< 0.001**
10 - 14	8 (20.5)	86 (50.9)	1.0	
**Ethnic Group:**				
Yoruba	20 (51.3)	43 (35.5)	3.3(1.45 – 7.52)	**0.003**
Igbo	11 (48.7)	78 (64.5	1.0	
**Age at Diagnosis (years):** (Mean ± SD)	3.0 **±** 2.8	3.3 **±** 2.8		0.665
**Baseline BMI/Weight for Age Z Score** Mean (± SD)	-1.22 (1.1)	-0.98 (1.3)		0.310
**WHO Clinical Stage:** Mean (± SD)	2.4 (0.8)	2.2 (0.9)		0.425
**ART (n = 202):**				
1^st^ Line	32 (82.1)	136 (83.4)	0.91 (0.36 – 2.27)	0.835
2^nd^ Line	7 (17.9)	27 (16.6)	1.0	
**ART Duration (years):** (Mean ± SD)	6.9 (2.72)	6.13 (2.99)		0.248

**Table 4:** comparison of clinical characteristics amo ng participants with SCT and HbAA

Characteristic	SCT (n = 39)	HbAA (n =169)	OR (95% CI)	P value
**History of Blood Transfusion:**				
Yes	3 (7.7)	22 (13.0)	0.55 (0.16-1.96)	0.356
No	36 (92.3)	147 (87.0)	1.0	
**History of Jaundice:**				
Yes	3 (7.7)	7 (4.1)	1.93 (0.47-7.82)	0.350
No	36 (92.3)	162 (95.9)	1.0	
**History of Bone/Joint Pains:**				
Yes	1 (2.6)	6 (3.6)	0.73 (0.08-6.24)	0.772
No	37 (97.4)	162 (96.4)	1.0	
**History of TB**:				
Yes	22 (56.4)	40 (24.1)	4.08 (1.97-8.43)	< 0.001
No	17 (43.6)	126 (75.9)	1.0	

**Table 5:** baseline and current virologic and haematologic parameters among participants with SCT and HbAA

Characteristic	Baseline			Current		
	SCT (n = 39)	HbAA (n =169)	P value	SCT (n = 39)	HbAA (n =169)	P value
**Viral Load (log):** Mean (± SD)			**0.048**			0.345
	415,384	812,764		11,656.5	42,834.6	
	(545,026.7)	(1744181.6)		(34,152.9)	(333,679.0)	
**CD4 count:** Mean (± SD)			0.468			0.557
	870.1	894.8 (717.4)		1,004.0	1,058.1	
	(579.3)			(561.2)	(516.8)	
**Hb:**Mean (± SD)	9.0 (1.7)	9.5 (1.4)	0.319	11.2 (1.1)	11.3 (1.2)	0.470

## Discussion

This study showed a sickle cell trait (SCT) and sickle cell disease (SCD) prevalence of 18.8% and 0.0% respectively. There was no sex predilection for SCT. However, SCT was significantly associated with being of Yoruba ethnic group, having lower HIV RNA viral load, and having HIV/TB coinfection. The zero prevalence of SCD and 18.8 prevalence of SCT from this study are lower than the reported prevalence of both conditions from Nigeria and sub-Saharan Africa of 2-3% and 25-30% respectively [9-12]. This disparity in prevalence rates could be due to the fact that both SCD and HIV cause significant paediatric mortality, with some reports suggesting childhood mortality rates in excess of 50% among children born with SCD [25, 26] and HIV accounting for 3-4% of under-five mortality in SSA [27]. It has also been reported that more than 50% of HIV-infected children die within the first 2 years of life without antiretroviral therapy (ART) [28]. It thus may be that children with HIV and SCD comorbidity, especially those perinatally infected with HIV succumbed at an early age to the dual burden of disease and so had no opportunity to be enrolled into the HIV treatment programme. The possible early demise of children with SCD/HIV comorbidity does not however explain the lower prevalence of the carrier state of the sickle cell gene i.e. the sickle cell trait, in our study population. The possible detrimental effect of the sickle cell gene on HIV [16, 18] may be responsible for this finding. Thus on exposure to HIV either during or after the perinatal period, the virus may be effectively cleared by the immune system of a person with sickle cell trait so that HIV infection is not established in them in the same proportion as in the general population. Though the lower prevalence of sickle Hb in this study pertains to the sickle cell trait as opposed to sickle cell disease, it is similar to the results of a cross-sectional study carried out in the United States which examined co-morbidities of SCD and HIV infection [14]. The authors also found that the frequency of HIV infection was significantly lower in patients with SCD than in those without SCD (1.5% versus 3.3%). There have also been reports of a higher frequency of HIV long-term nonprogressors in persons with HIV/SCD comorbidity than in persons with HIV alone [24].

Though there are very few studies assessing sickle cell trait and HIV infection, Kumari *et al* [23] in their study among African-Americans also found a reduced prevalence of HIV among persons with HbAS compared to those with HbAA. They also found similar Hb levels but significantly lower HIV viraemia in those with SCT, similar to the findings in this study. The lower HIV viraemia was postulated to result from the effect of iron metabolism and hypoxia on some HIV-1 replication factors. William and colleagues [29] in their study on hypoxia and HIV replication and latency discovered that hypoxia increased expression of Hypoxia Inducible Factors (HIFs) and reduced replication of HIV-1. The significant hypoxia which can occur in SCT [8] may therefore be responsible for reduced replication of HIV-1 and thus lower viraemia. Being younger than 10 years and from the Yoruba ethnic group were significantly associated with SCT (p < 0.01). This finding is keeping with a study that reported the Yoruba ethnic group to have the highest prevalence of SCD in Nigeria [10]. Majority of the parents completed secondary school (66.8% and 65.8% for fathers and mothers respectively). This may also account for the zero prevalence of SCD seen in this study. People with secondary or higher education are more likely to be informed about SCD, know their Hb genotype before marriage, and refrain from marriage to somebody with SCT if they are also carriers of the sickle cell trait, thus eliminating the possibility of their having an offspring with SCD. Majority of the participants (58%) presented at an early stage of HIV disease (stage 1 and 2). This could be as a result of intensified provider initiated counselling and testing for prompt diagnosis before progression to severe forms of the disease. The study was carried out in a comprehensive HIV treatment facility where infected adults are counselled to bring in their children, as well as siblings of infected children, even apparently healthy ones, for HIV testing services. Thus a lot of these children are diagnosed when asymptomatic or with mild disease presentation. At enrolment into HIV care, majority of the children were anaemic (mean Hb = 9.4 /dl) and had high HIV RNA viral load. This is the usual finding in HIV-infected children, and HIV is also a recognized cause of anaemia in children [28]. There was a statistically significant difference between the baseline and current virologic and haematologic parameters of all study participants. Most of the study participants (97.1%) were on antiretroviral drugs (ARVs) and this finding buttresses the well-recognized efficacy of highly active antiretroviral therapy (HAART) which has turned a once lethal condition into a chronic and manageable one with prospects of perinatally infected children living into productive adulthood [30, 31]. This clinical improvement was evident in those with HbAA and those with SCT showing that SCT did not interfere with the response to HAART.

## Conclusion

The prevalence of sickle cell trait among HIV infected children was found to be low among the study cohort and none had sickle cell disease. The participants with SCT also had lower HIV viraemia than those with HbAA. This finding suggests that further studies are needed to elucidate how this can be applied to improve child health in Nigeria and Africa.

### What is known about this topic

- HIV infection and the sickle haemoglobin are prevalent causes of childhood morbidity and mortality in sub-Saharan Africa;
- There is a dearth of information on the interaction between HIV and the sickle cell gene in children in the sub region.

### What this study adds

- Our study found an 18.8% prevalence of sickle cell trait among HIV infected children;
- The HIV RNA viral load was significantly lower in the children with sickle cell trait compared to those with normal haemoglobin genotype.

## Competing interests

The authors declare no competing interests.
